# WHO systematic review of maternal morbidity and mortality: the prevalence of severe acute maternal morbidity (near miss)

**DOI:** 10.1186/1742-4755-1-3

**Published:** 2004-08-17

**Authors:** Lale Say, Robert C Pattinson, A Metin Gülmezoglu

**Affiliations:** 1 UNDP/UNFPA/WHO/World Bank Special Programme of Research, Development and Research Training in Human Reproduction, Department of Reproductive Health and Research, World Health Organization, Geneva, Switzerland; 2 MRC Maternal and Infant Health Care Strategies Research Unit, University of Pretoria, Kalafong Hospital, South Africa

## Abstract

**Aim:**

To determine the prevalence of severe acute maternal morbidity (SAMM) worldwide (near miss).

**Method:**

Systematic review of all available data. The methodology followed a pre-defined protocol, an extensive search strategy of 10 electronic databases as well as other sources. Articles were evaluated according to specified inclusion criteria. Data were extracted using data extraction instrument which collects additional information on the quality of reporting including definitions and identification of cases. Data were entered into a specially constructed database and tabulated using SAS statistical management and analysis software.

**Results:**

A total of 30 studies are included in the systematic review. Designs are mainly cross-sectional and 24 were conducted in hospital settings, mostly teaching hospitals. Fourteen studies report on a defined SAMM condition while the remainder use a response to an event such as admission to intensive care unit as a proxy for SAMM. Criteria for identification of cases vary widely across studies. Prevalences vary between 0.80% – 8.23% in studies that use disease-specific criteria while the range is 0.38% – 1.09% in the group that use organ-system based criteria and included unselected group of women. Rates are within the range of 0.01% and 2.99% in studies using management-based criteria. It is not possible to pool data together to provide summary estimates or comparisons between different settings due to variations in case-identification criteria. Nevertheless, there seems to be an inverse trend in prevalence with development status of a country.

**Conclusion:**

There is a clear need to set uniform criteria to classify patients as SAMM. This standardisation could be made for similar settings separately. An organ-system dysfunction/failure approach is the most epidemiologically sound as it is least open to bias, and thus could permit developing summary estimates.

## Background

Severe acute maternal morbidity (SAMM), also known as "near miss", is defined as "A very ill pregnant or recently delivered woman who would have died had it not been that luck and good care was on her side" [1,2]. This concept is relatively new in maternal care, but is increasingly becoming important in areas with low maternal mortality ratios or where the geographic area is small [3,4]. The use of data collected on SAMM has been shown to be a mechanism for identifying health system failures or priorities in maternal health care more rapidly than maternal deaths [5]. It has the advantage of events still being rare enough not to overload clinicians and data capturing personnel within a facility. Its routine use as an indicator, however, is limited due to the lack of uniform criteria of identification of the cases.

This study was undertaken to systematically review all available studies on SAMM with a view to establishing the global prevalence and examining the usefulness as a maternal health indicator.

## Methods

This study is a part of a bigger systematic review undertaken by the UNDP/UNFPA/WHO/World Bank Special Programme of Research, Development and Research Training in Human Reproduction (HRP), Department of Reproductive Health and Research at the World Health Organization (WHO) to obtain prevalence/incidence data on maternal mortality and a range of morbidities including SAMM. The methodology of the systematic review followed an a priori protocol and involved an extensive search of all relevant published/unpublished data from 1997 to 2002. The methodology of the systematic review and the search strategy have been described elsewhere [6]. In brief, we searched 10 electronic databases, WHO regional databases, internet and reference lists, contacted experts in the field, and hand-searched relevant articles in the WHO Library. Criteria for inclusion of studies in the review were: inclusion of data relevant to pre-defined conditions, specified dates for data collection period, including data from 1990 onwards, sample size >200 and a clear description of methodology.

A data extraction instrument was used to extract data from included studies. This instrument includes 48 items distributed in five modules three of which were relevant to this analysis. Modules were designed to collect information on (i) the general study level characteristics such as design, population, setting, (ii) prevalence/incidence of maternal morbid conditions, and (iii) quality assessment of morbidity reports. Reporting of definitions and of the procedures used for identification of cases for morbidities were part of quality assessment. We did not assign quality scores to articles, but preferred to present available information on variables regarded as quality components (including reporting of definitions, case-identification criteria, characteristics of setting and participants).

Nearly 65 000 reports were screened initially by titles and/or abstracts of which more than 4500 were retrieved for full-text evaluation. More than 2500 of these were included in the review. Data extracted were entered into a specifically constructed database and tabulated using SAS software.

A small number of the articles in the review report on SAMM, near miss or a similar definition such as severe morbidity, critically ill obstetric patient. Although we included other articles reporting on individual severe morbidities within their own category of conditions (e.g. severe hypertension within hypertensive disorders of pregnancy), this particular article is concerned with those papers which define a separate entity of SAMM or similar, or those which report on the most recognised end-points for SAMM (i.e. emergency hysterectomy and obstetric admissions to intensive care units).

We later conducted an updated search of MEDLINE and Popline using the keywords 'near miss morbidity' and 'severe maternal morbidity' to find out about any recent publications that could be of importance, but were not included in the database due to the end date of the original search (2002) for the bigger systematic review. The date of this complementary search was June 2004.

We describe below the included studies with an emphasis on the different definitions used and criteria for identification of the cases.

## Results

A total of 30 reports of SAMM are included in the systematic review (twenty-seven identified by the original search and three by the complementary search). Study designs are mainly cross-sectional and most of them are conducted in tertiary care hospitals (see Additional file 1). Most of the studies describe the characteristics of the setting and participants as well as reporting definitions and procedures for identification of the cases.

There are essentially two types of definition of severe acute maternal morbidity; one describing what the authors meant by a near miss; and the other describing a response to an event such as hysterectomy or admission to intensive care unit (ICU). Fourteen studies define a specific SAMM or near-miss 'condition' [1,2,7-18] while nine consider admissions to ICU as near-miss cases [19-27] and the remainder report on emergency hysterectomies [28-34].

In the majority of articles there is an intuitive agreement on what a near miss means – a woman who almost died but survived. Identification of cases, however, is complex and varies widely across studies. We listed the articles in Additional file 1 in three categories according to criteria used to classify patients as being near miss; disease-specific (specified criteria for common conditions, e.g. pre-eclampsia, haemorrhage); management-specific (specified criteria related to response to disease, e.g. hysterectomy or admission to ICU); and organ-system dysfunction/failure based (specified criteria for dysfunction or failure related to each organ system). One study reports the proportion of the admissions to ICU separately as well as the total number of SAMM cases [8]. We included this report under the section of organ-system based identification criteria.

Individual prevalence rates for SAMM vary between 0.80% – 8.23% in the first category of studies that use disease-specific criteria. Case-finding criteria differ significantly within this category as well. In addition, some of the studies with similar criteria use mainly clinician's evaluation for identification [15], while others have established threshold levels for the degree of severity of the conditions of concern [7,18]. This is probably due to contextual factors such as the availability of facilities with sufficient diagnostic tools.

The range is 0.38% – 1.09% in the group of reports that use organ-system based criteria and include unselected women. Rates are lower (0.01% – 2.99%) and variation is lesser in the category of studies using management-based criteria.

An expected finding is the difference between resource-poor and more advantageous settings in the prevalence of SAMM. In resource-poor settings, 4–8% of pregnant women who deliver in the hospitals will experience SAMM when case-identification criteria are based on specific diseases. This rate is around 1% when the organ failure is considered. In more developed country settings, the rates are around 1% with disease-specific and 0.4% with organ-system based criteria, respectively. The results also suggest that the use of organ-system based criteria is more specific in identifying the real SAMM cases.

## Discussion

Due to the wide variation in identification of the cases, it is not possible to pool data and make a summary estimate for SAMM. Because of the variation in case identifications in the three categories of identification criteria as well as variation within each category (e.g. for disease-specific criteria – the use of physician's evaluation versus technology requiring tests) it is difficult to make comparisons as well. Nevertheless, it is evident that the prevalence of SAMM is higher in studies conducted in less developed country settings. Rates seem to be higher also in studies that use disease-specific criteria as compared to those using organ-system based criteria for similar settings. This finding suggests less specificity of disease-specific criteria in identifying real SAMM cases.

Although less specific, the use of disease-specific criteria has some advantages; it is easy to interpret, cases can be identified retrospectively, and the quality of care for that particular disease can be assessed [18,35]. However, the approach concentrates on certain diseases, and thus, other problems such as pulmonary embolus, which is an important cause of maternal death in developed countries could be ignored [36]. In addition, definition of conditions may not always be straightforward. For example, the same threshold for severe haemorrhage could have different consequences in women with normal haemoglobin levels or those with severe anaemia. Furthermore, although detailed objective criteria are established for case identification in developed country settings [18], the limited availability of resources in less developed settings may not permit this level of detail. Therefore, identification of cases is likely to be less accurate when the diagnosis depends on clinical estimates [2,15]. Use of management-specific criteria is advantageous in that it is simple to identify the cases, but it depends on many other variables such as the availability of ICU beds, the facilities in an ICU, or differing views about and indications for hysterectomy. Also, the approach does not include all SAMM cases. One study reporting SAMM according to organ-system based criteria and admissions to ICU separately shows that admissions to ICU represent only one third of all SAMM cases [8].

Use of organ-system based criteria allows for identifying all severe morbidities and then investigating the primary cause, thus does not discard any particular condition. It is the most reproducible across similar areas and criteria can be defined according to resources available. High technology requiring laboratory and haemodynamic investigations can be avoided. However, it is the most labour-intensive for identifying cases, hence criteria for inclusion as near miss must be strict. Bias can be introduced if data collection is incomplete and prevalence can be underestimated.

Two approaches are used as potential methods of assessing the care SAMM cases receive. "Mortality Index-MI" is defined as the ratio of maternal deaths among the SAMM cases to the sum of maternal deaths and SAMM cases [35,37]. It represents the proportion of women who presents with a SAMM and subsequently dies [37]. Another approach is to calculate the ratio of SAMM to mortality [8,18]. We attempted to calculate the ratio of SAMM to mortality for all studies included under the categories of disease-specific or organ-system based case identification criteria (see Additional file 1). We did not calculate MI because it was not clear from some reports whether reported maternal deaths were identified as SAMM or not prior to death.

It is clearly illustrated in the studies that more SAMM cases are likely to die in resource-poor settings than in more developed country settings. For example, the studies conducted in Niger, Benin and Malaysia give the morbidity to mortality ratio as 11–12 [10,15,17] while this is 117–223 in studies conducted in Europe [11,18] in the category where disease-specific criteria are used. The same applies to the category of organ-system based criteria; morbidity: mortality ratio is 5–8 in South Africa [1,14,15] and 49 in Scotland [8]. These findings suggest that an indicator that relates SAMM to maternal deaths could be a useful method in assessing the care SAMM cases receive. However, the definitions and identification of cases should be standardised at least for similar settings and the indicator needs to be clearly defined.

## Conclusion

Considering all complexities in definition and case-identification of SAMM, it is necessary that studies clearly describe their identification criteria for the cases. There is a clear need to set criteria to identify SAMM cases. Use of organ-system based criteria seems to be a more useful approach in identifying cases as variation in defining criteria can be avoided, particularly for similar settings. It would then be easier to establish summary estimates for SAMM prevalence which could serve as a measure of maternal health and quality of care indicator.

## Competing interests

None declared.

## Authors' contributions

AMG and LS coordinated the conduct of the systematic review of maternal morbidity and mortality. RCP and LS outlined the manuscript. LS reviewed the studies and wrote the initial draft. AMG and RCP substantially improved the manuscript.

## Supplementary Material

Additional file 1 Table describing important variables of all studies included in the systematic review Click here for file
