# Supplementary material for: WHO systematic review of maternal morbidity and mortality: the prevalence of severe acute maternal morbidity (near miss)
Source: Reprod Health. 2004 Aug 17;1:3. doi: 10.1186/1742-4755-1-3 (PMC516581; doi:10.1186/1742-4755-1-3)
Supplement: Additional file 1 — Table describing important variables of all studies included in the systematic review [file 1742-4755-1-3-S1.doc]

Table 1. Included studies according to case-identification criteria

| **Study** | **Country and years** | **Study design** | **Setting and participants[[1]](#footnote-2)*** | **Notes on case identification** | **N of cases** | **Sample size** | | **Prevalence %** | **N of deaths** | **Morbidity/ mortality** |
| --- | --- | --- | --- | --- | --- | --- | --- | --- | --- | --- |
| **Disease-specific criteria** | | | | | | | | | | |
| Prual 1998[15] | Niger | Cross sectional prospective | Maternity / deliveries | Eight groups of conditions are defined. Clinical evaluation by an experienced physician | 232 | 4081 | | 5.68 | 21 | 11 |
| Filippi 1998[10] | Benin  1995-96 | Cross sectional retrospective | Teaching/ deliveries | According to developed algorithms, based on specific conditions such as eclampsia, haemorrhage and infections | 353 | 4291 | | 8.23 | 30 | 12 |
| Sivalingam 1999[17] | Malaysia  1996 | Cross sectional retrospective | Maternity / deliveries | Three groups of conditions - severe hypertensive disorders, massive haemorrhage, life-threatening complications (includes organ failures and admissions to ICU) | 122 | 9933 | | 1.23 | 10 | 12 |
| Prual 2000[2] | Multicountry West Africa 1994-96 | Cohort prospective | Population based/ live births | Includes specified conditions (haemorrhage, dystocia, hypertension, sepsis), hysterectomies, c/s. Excludes abortion. Mainly clinical evaluation | 1174[[2]](#footnote-3) | 19 694 | | 6.17[[3]](#footnote-4) | 41 | 29 |
| Khosla 2000[13] | India  1998 | Cross sectional retrospective | Hospital - not specified/ deliveries | Based on individual conditions such as hypertension, haemorrhage, abortion, infections. No defined criteria for severity | 224 | 5124 | | 4.37 | 31 | 7 |
| Waterstone 2001[18] | UK  1997-98 | Case-control retrospective | Population based/  deliveries | Based on conditions including severe pre-eclampsia, eclampsia, HELLP, severe haemorrhage, severe sepsis, uterine rupture according to established criteria | 588 | 48 865 | | 1.20 | 5 | 117 |
| Girard 2001[11] | France  1995 | Cross sectional retrospective | Population based/ deliveries | Specific conditions - haemorrhage, hypertension and sepsis, established criteria for the degree of severity | 223 | 27 872 | | 0.80 | 1 | 223 |
| Bouvier-Colle 2001[7] | Multicountry  Europe  1995-96 | Cross sectional retrospective | Population based/ deliveries | Includes specific conditions - severe haemorrhage, hypertension and sepsis, established criteria for the severity | 1843 | 182 589 | | 1.01 | NR | NA |
| **Organ-system based criteria** | | | | | | | | | | |
| Mantel 1998[1] | South Africa 1996-98 | Cross sectional (audit) prospective | Teaching/  deliveries | Organ-system based criteria (Mantel criteria) | 147 | 13 429 | | 1.09 | 30 | 5 |
| Pattinson 2002[14] | South Africa  2000 | Cross sectional (audit) prospective | Teaching/ deliveries | Organ-system based criteria (Mantel criteria) | 121 | 13 854 | | 0.87 | 26 | 5 |
| Cochet 2003[9] | South Africa  2001 | Cross sect ional (audit) prospective | Teaching/ deliveries | Organ-system based criteria (Mantel criteria) | 131 | 15 978 | | 0.82 | 16 | 8 |
| Kaye 2003[12] | Uganda  2000 | Cross sectional retrospective | Teaching/ obstetric emergencies | Organ-system based criteria (Mantel criteria) | 87[[4]](#footnote-5) | 980 | | 10.61[[5]](#footnote-6) | 17 | 5 |
| Brace 2004[8] | Scotland  2001-02 | Cross sectional prospective | All maternity units/  deliveries | Organ-system based criteria (Mantel criteria), diagnosis by pathophysiological features rather than clinical experience | 196 | 51 165 | | 0.38 | 4 | 49 |
| ***Mixed criteria (disease-specific and organ-system based)*** | | | | | | | | | | |
| Sahel 2001[16] | Morocco  1998 | Cross sectional retrospective | General/ deliveries | A mixture of disease-specific, organ-system based and management-based criteria | 76 | 5686 | | 1.34 | 5 | 15 |
| **Management-specific criteria** | | | | | | | | | | |
| ***Emergency hysterectomy*** | | | | | | | | | | |
| Gould 1999[30] | UK  1992-98 | Cross sect ional (audit) retrospective | General/ deliveries | Massive postpartum haemorrhage (PPH) and abnormal placentation - main reasons | 10 | 22 240 | 0.04 | | - | NA |
| Nasrat 1999[32] | Saudi Arabia 1990-97 | Cross sectional retrospective | General/ deliveries | Severe PPH failed to response to medical interventions, prolonged labour | 23 | 18 842 | 0.12 | | 1 | NA |
| Bakshi 2000[29] | US  1990-95 | Cross sectional retrospective | Teaching/ deliveries | Mainly due to PPH originating from placental anomalies | 39 | 14 220 | 0.27 | | - | NA |
| Alyasali 2000[28] | Saudi Arabia 1990-98 | Cross sectional retrospective | Maternity/ deliveries | Mainly due to PPH originating from placental anomalies | 29 | 74 200 | 0.04 | | 2 | NA |
| Yamamoto 2000[34] | Japan  1985-98 | Cross sectional retrospective | Referral/ deliveries | Leading causes – uterine rupture, dissemine intravascular coagulation, uterine atony | 17 | 118 626 | 0.01 | | NR | NA |
| Wenham 2001[31] | UK  1985-97 | Cross sectional retrospective | Teaching/ deliveries | Main reasons, uterine rupture, PPH, placenta praevia | 20 | 53 312 | 0.04 | | 1 | NA |
| Noor 2001[33] | Pakistan  1995 | Cross sect retrospective | Teaching/ deliveries | Mainly due to uterine rupture, atony, laceration, placental anomalies | 88 | 2940 | 2.99 | | 15 | NA |
| ***Admission to ICU*** | | | | | | | | | | |
| Bewley  1997[21] | UK  1991-1992 | Cross sect retrospective | Teaching/ deliveries | Mostly due to haemorrhage (>2000 ml) (n=14) and hypertension (n=12) | 30 | 6039 | 0.49 | | 2 | NA |
| Bouvier-Colle  1997[22] | France  1991-1992 | Cross sectional retrospective | All intensive care units in three regions/ live births | Mostly due to hypertension (26%), haemorrhage (20%) and indirect obstetric causes (17%) | 435 | 140 323[[6]](#footnote-7) | 0.31 | | 22 | NA |
| Baskett 1998[19] | Canada  1980-93 | Cross sectional retrospective | General/ deliveries | Women requiring transfer to two adjacent units. Ectopic pregnancies and abortions are not included in the hospital population | 55 | 76 119 | 0.08 | | 1 | NA |
| Rodriguez Iglesias 1999[26] | Cuba  1987-98 | Cross sectional retrospective | Teaching/ deliveries | NR | 52 | 21 510 | 0.24 | | NR | NA |
| Ryan 2000[27] | Ireland  1996-98 | Cross sectional retrospective | Maternity/ deliveries | Mostly due to haemodynamic instability (85%), 45% had pre-eclampsia as primary diagnosis. | 123 | 12 070 | 1.02 | | - | NA |
| Loverro 2001[24] | Italy  1988-98 | Cross sectional retrospective | Teaching/ deliveries | NR | 41 | 23 000 | 0.18 | | 2 | NA |
| De Souza 2002[23] | Brazil  1991-2000 | Cross sectional retrospective | Teaching/ deliveries | 41% due to pre-eclampsia. | 40 | 28 660 | 0.14 | | NR | NA |
| Murphy 2002[25] | UK  1988-99 | Cohort retrospective | Teaching/ deliveries | Mainly cardiac, hypertensive and haemorrhagic indications. | 50 | 51 576 | 0.10 | | 3 | NA |
| Ben Letaifa 2002[20] | Tunisia  1998-2000 | Cross sectional retrospective | General/ deliveries | Indication - requirement of controlled ventilation | 20 | 24 812 | 0.08 | | 6 | NA |

NR: not reported

NA: not applicable

1. ^^ Type of the setting and type of the denominator used [↑](#footnote-ref-2)
2. Recalculated by separating maternal deaths (n=41) from reported severe morbidities (n= 1215) [↑](#footnote-ref-3)
3. 1215/19 694 [↑](#footnote-ref-4)
4. Recalculated by separating maternal deaths (n=17) from reported severe morbidities (n= 104) [↑](#footnote-ref-5)
5. 104/980 [↑](#footnote-ref-6)
6. Calculated using the rate given (310 per 100 000 livebirths) [↑](#footnote-ref-7)
